# Multiple Cholecystoenteric Fistulae With Bouveret Syndrome and Acute Pancreatitis: A Rare Combination

**DOI:** 10.7759/cureus.38152

**Published:** 2023-04-26

**Authors:** Dattaprasanna R Kulkarni, Pooja P Goradia, Neha D Kulkarni, Shrikant Garge

**Affiliations:** 1 Gastrointestinal Surgery, Lilavati Hospital & Research Center, Mumbai, IND; 2 Gastrointestinal Surgery, Liver & Pancreas Clinic, Mumbai, IND; 3 Medicine, K. J. (Karamshi Jethabhai) Somaiya Medical College, Mumbai, IND

**Keywords:** minimally invasive surgery, laparoscopy, endoscopy, acute pancreatitis, bouveret syndrome

## Abstract

Multiple cholecystoenteric fistulae, Bouveret syndrome (a form of gallstone ileus), and acute pancreatitis occurring together is very rare. Diagnosis is seldom clinical and is mostly based on computerised tomography (CT) or magnetic resonance imaging (MRI). Endoscopy and minimally invasive surgery have revolutionised the treatment of Bouveret syndrome and cholecystoenteric fistula, respectively, over the last two decades. Laparoscopic repair of cholecystoenteric fistula followed by cholecystectomy is successful on a consistent basis with skilled laparoscopic suturing and advanced laparoscopy. In patients with Bouveret syndrome, when the stone is <4cm and is in the proximal duodenum, it is usually amenable for endoscopic extraction with snares, nets, forceps and lithotripsy. When endoscopy is unavailable or fails, laparoscopic surgery is suitable for these patients. However, stones >4 cm, located in the distal duodenum, multiple fistulae, and associated acute pancreatitis may necessitate open surgery. We present here a case of a 65-year-old Indian female with multiple cholecystoenteric fistulae and Bouveret syndrome with acute pancreatitis with a 6.5 cm gallstone diagnosed on CT scan and MRI and treated successfully by open surgery. We also review the current literature on the management of this complex problem.

## Introduction

Cholecystoenteric fistula (CEF) is a rare and late complication of calculous cholecystitis [1,2]. Cholecystoduodenal fistula (CDF) is the most common (75-80%) followed by cholecystocolic fistula (CCF) and cholecystogastric fistula (CGF) [3]. Coexistence is uncommon [4]. CDF mostly presents with chronic complaints [5]. In rare cases, it can present with acute intestinal obstruction by migrated gallstones, also called gallstone ileus, which is reported in 0.3-0.5% of patients with gallstones [6]. Bouveret syndrome (BS) is considered a type of gallstone ileus, wherein the gallstone causes duodenal or gastric outlet obstruction after passing through a fistula with duodenum or stomach, respectively [6,7]. It is seen in 1-3% of patients with gallstone ileus [6]. The incidence of CCF is 0.06-0.14% [4]. BS with CCF is rarely reported [8]. BS with acute pancreatitis has also been rarely reported [9]. We present here a case of so far unreported combination of multiple CEFs with BS and acute pancreatitis. We also discuss the current trends in the management of this complex problem.

## Case presentation

A 65-year-old Indian female presented with abdominal pain, bloating, nausea, and vomiting for the past seven days. Abdominal pain was localised to upper abdomen, continuous and dull in nature with occasional intermittent colic. There were multiple episodes of vomiting, which was projectile and non-bilious in nature. The patient was constipated but was passing flatus. She did not have pale stools, dark urine, fever, or weight loss. She had never consumed alcohol. The patient was diabetic and hypertensive for 15 years; both were under control with medications. She was afebrile. Her heart rate was 120 beats/minute and blood pressure was 90/60 mmHg. Her abdomen was distended and had generalised tenderness. Bowel sounds were absent. Hernia orifices were normal. Her blood investigations at the time of admission are shown in Table 1.

**Table 1:** Blood investigations at the time of admission.

Test	Result	Normal reference range
Haemoglobin	10.5 gm/dl	12.0-15.0 gm/dl
Total white cell count	18320/cu.mm	4000-10000/cu.mm
C-reactive protein	236 mg/L	<5.0 mg/L
Serum amylase	316 U/L	28--100 U/L
Serum lipase	264 U/L	13-60 U/L
Serum bilirubin total	0.5 mg/dl	<1.2 mg/dl
Aspartate amino transferase (SGOT)	18 IU/L	0-32.0 IU/L
Alanine amino transferase (SGPT)	12 IU/L	<33 IU/L
Gamma-Glutamyl transferase (GGT)	30 IU/L	5.0-36.0 IU/L
Alkaline Phosphatase (ALP)	88 IU/L	35.0-105.0 IU/L

**Figure 1:**
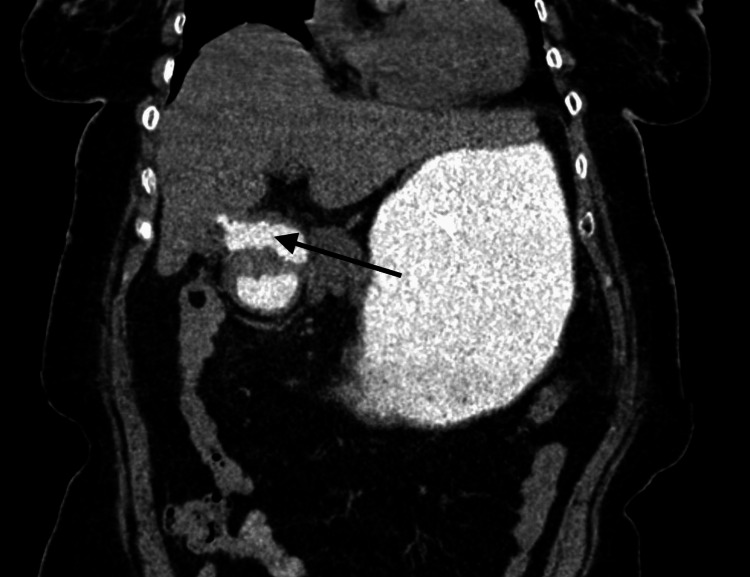
CT scan showing cholecystoduodenal fistula (black arrow)

**Figure 2:**
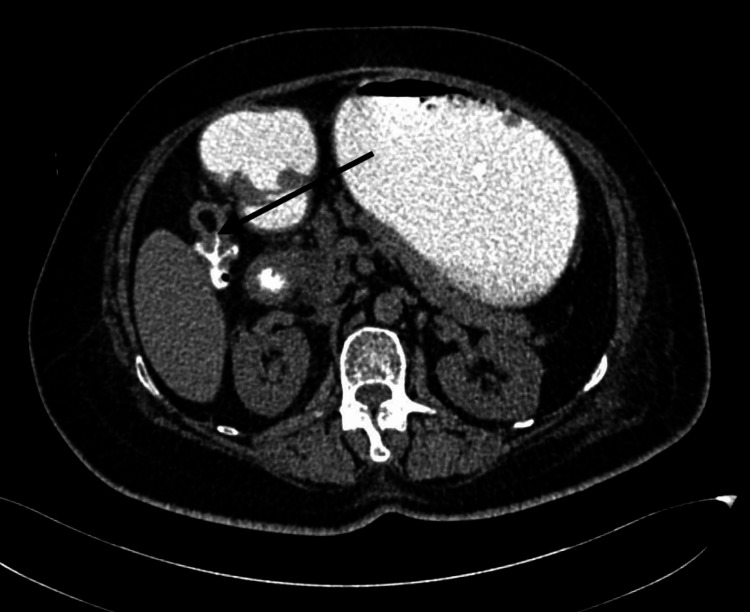
CT scan showing cholecystocolic fistula (black arrow)

**Figure 3:**
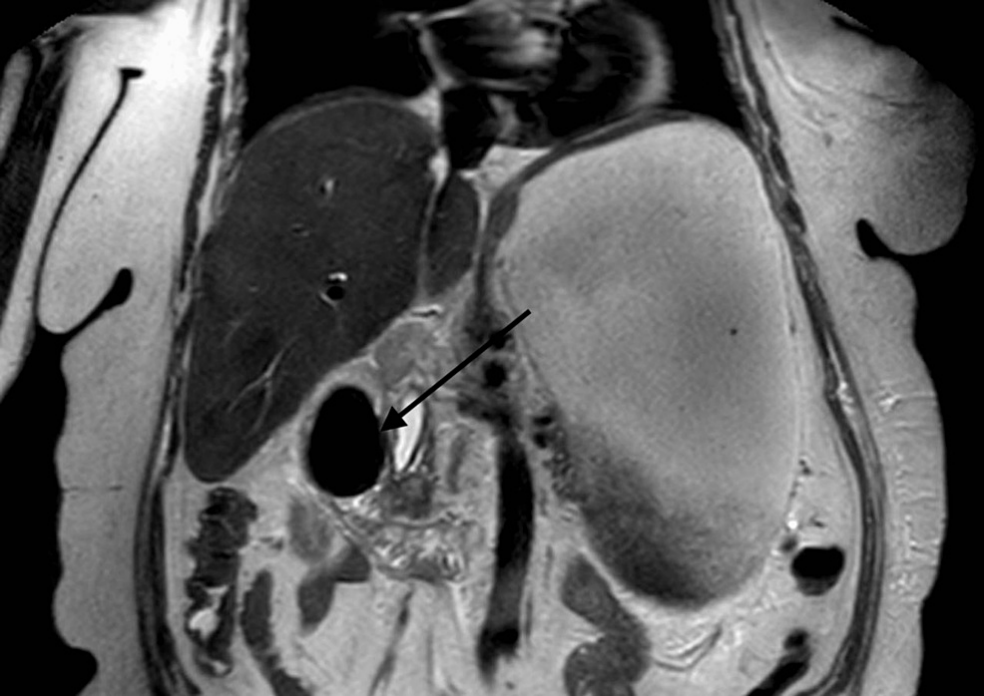
MRI showing giant stone in second part of duodenum (black arrow) and massively distended stomach

Ultrasonography (USG) of the abdomen showed a solitary gallstone. CT scan of the abdomen with oral and intravenous contrast and MRI/magnetic resonance cholangiopancreatography (MRCP) showed a 6 cm calculus in the second part of the duodenum, air in the gall bladder and biliary tree, CDF, dilated stomach, oedematous acute pancreatitis, and CCF (Figures 1-3).

**Figure 4:**
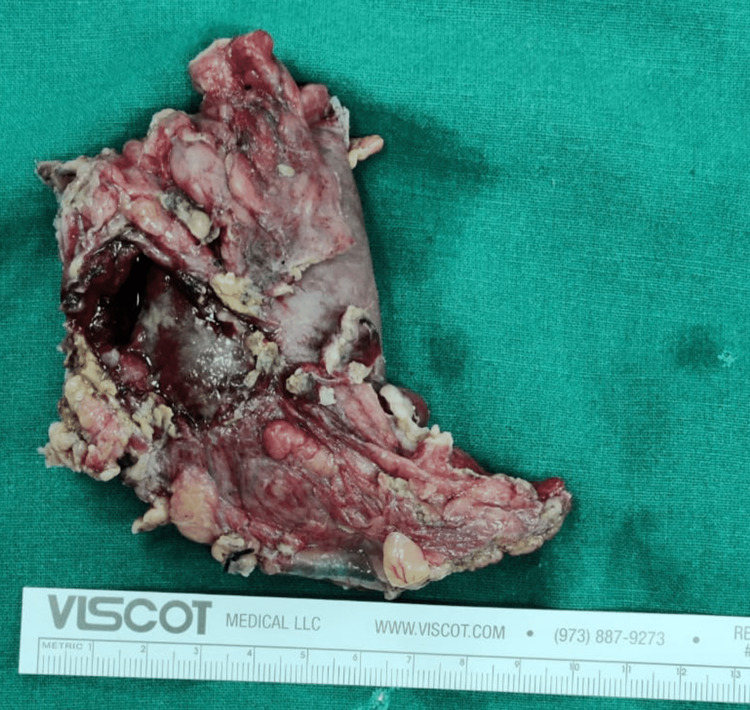
Specimen of antroduodenectomy showing distal antrum with proximal duodenum and opening of the fistula tract in the anterior wall of duodenum

Since we had reached the diagnosis on imaging, a diagnostic gastroscopy was not done. In view of the size of the stone, the gastroenterologist/endoscopist decided not to attempt endoscopic retrieval and instead suggested surgery. Laparoscopy revealed dense adhesions between the gall bladder, colon, and duodenum. Hence, a laparotomy through a right subcostal incision was done. After adhesiolysis, CCF was closed with stapler with a small margin of colonic wall. Stone was densely impacted in the fistulous tract and the second part of the duodenum. Due to the impaction, orientation, and size, the stone couldn’t be manoeuvred towards the pylorus. The second part of the duodenum with the stone and inflamed pancreas/peripancreatic tissues had become a phlegmon that prevented safe duodenal Kocherization for duodenotomy on the second part of the duodenum. Hence, a pylorotomy was done to dislodge the stone from within. However, that too was unsuccessful and the pylorotomy had to be extended onto the first part of the duodenum and finally onto the fistulous tract. The stone was extracted through this pyloroduodenotomy. It could not be closed transversely or longitudinally as the tissue was friable, unhealthy, and was not holding the sutures well. Also, there was the need to remove the entire fistula tract though the frozen section examination had not shown any malignancy. Therefore an antroduodenectomy (including fistula) was done followed by Billroth II reconstruction. Figure 4 shows the specimen of antroduodenctomy.

**Figure 5:**
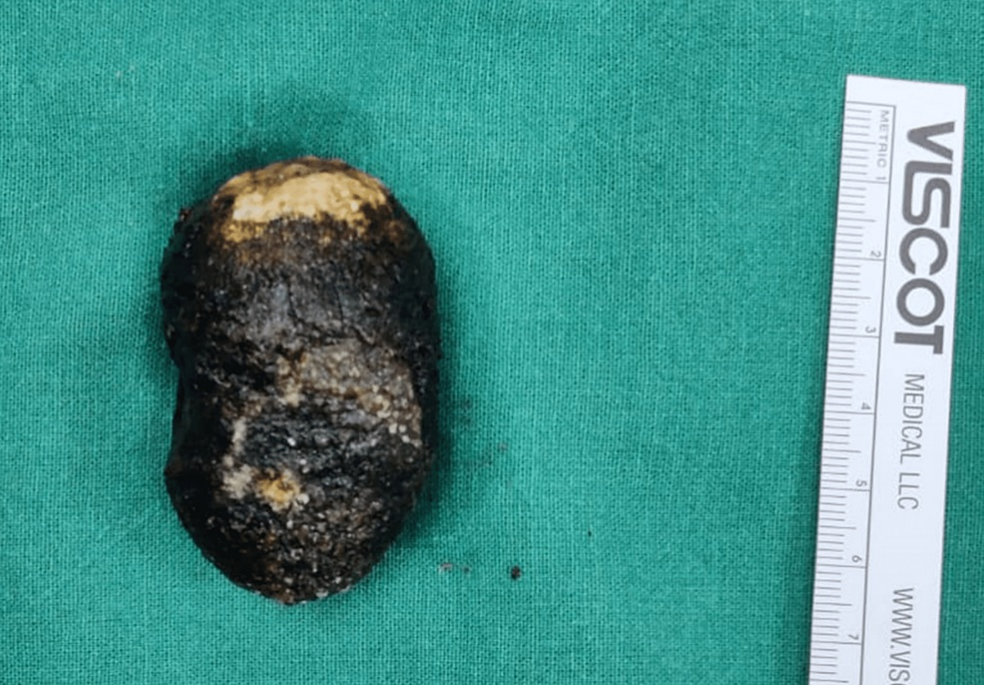
Stone (6.5 cm) removed from the cholecystoduodenal fistula and second part of duodenum

Surgery was completed after the cholecystectomy. The stone size on gross examination was 6.5 cm (Figure 5).

The patient recovered well except for a small volume duodenal stump leak on the seventh postoperative day through the drain placed during surgery. It settled with conservative treatment (parenteral nutrition and antibiotics) by the 18th postoperative day. She was discharged on the 20th postoperative day. Histopathology showed chronic cholecystitis. She was asymptomatic at the time of postoperative follow-ups at one and three months.

## Discussion

The incidence of CEF in cholelithiasis has significantly dropped (0.5-0.9%) due to the high number of laparoscopic cholecystectomies at a young age [1,5]. But the proportion of gallstone ileus is the same due to the large ageing population [6,10]. CEF and BS are predominantly seen in elderly females having longstanding gallstones [1,9,11]. Chronic inflammation and pressure of gallstone lead to fistula formation with surrounding organs [12,13]. Multiple fistulae are, however, rare; known associations being CDF with CCF followed by CDF with CGF, and Mirizzi syndrome and CDF [1-5]. 

Symptoms of CEF are nonspecific like right hypochondriac pain, nausea, and vomiting [1]. Chronic diarrhoea is a key complaint of CCF [4]. Smaller stones after escaping through CEF may cause recurrent subacute obstruction, while larger ones (>2.5 cm) can cause BS leading to abdominal pain, distension, and vomiting as in this case [11]. Acute pancreatitis is very rare with BS [9]. Only five cases have so far been reported [9,14-17]. Pressure from the impacted stone on the ampulla probably causes obstructive pancreatitis [16], as seen in our patient too.

Diagnosis of CEF or BS is rarely preoperative. Abdominal x-ray showing Rigler triad (pneumobilia, bowel obstruction, and an out-of-place gallstone) or tetrad (shifting position of the stone), barium study, USG, and endoscopy are inconsistent in diagnosis (10-60%) [2,11,18,19]. USG may miss the diagnosis because gall bladder is collapsed and air filled, stone is interpreted in gall bladder alone, or fistula tract is confused with bile duct [18]. Endoscopy may show duodenal obstruction while a stone embedded in the mucosa is missed [19]. Endoscopic retrograde cholangiopancreatography (ERCP) for choledocolithiasis and colonoscopy for diarrhoea may pick up an incidental CCF [4]. CT and MRI, however, perform better [2,11,18]. CT has 93% sensitivity, 100% specificity, and 99% accuracy [6,18]. It can still miss isoattenuating calculi [18]. Inflammation, fistula, isoattenuating stone, and choledocholithiasis are better appreciated by MRI [18]. Atrophied, thick-walled gall bladder with air, pneumobilia, poorly defined plane between the gall bladder and adjoining viscera, contrast opacification of fistula, and obvious stones are important findings in CT/MRI [2,18]. CT and MRI together picked up BS, acute pancreatitis, and CCF in our patient. It shows their efficacy to pick up multiple fistulas, a big preoperative advantage.

Treatment for BS was a one- or two-stage open surgery till the first endoscopic stone extraction in 1985 [6]. Since then, the success rate for endoscopy has gradually improved to almost 40% using snare, net, basket, and lithotripsy (mechanical and laser) [20-22]. It has reduced treatment-associated morbidity and mortality too [22]. A tool devised by Ong et al. based on stone length, location of stone impaction in duodenum, and number of endoscopic modalities to be used to predict the outcome of endoscopy showed excellent correlation [21]. Post hoc analysis by Swift et al. confirmed that the aforementioned tool can be used successfully to predict endoscopy outcomes in BS [22]. According to their analysis, stone ≤ 4 cm and in the proximal duodenum (first part) has higher success with endotherapy. For larger stones and those located in difficult locations, second-line surgery is needed in >60% of cases [22]. We avoided endoscopy for the same reason (stone size). However, stones up to 7 cm or located in the distal duodenum have been removed through endoscopy [20]. 

Surgery for BS should now be reserved where endoscopy infrastructure/expertise is unavailable, endoscopy has failed, the stone is >4 cm, or when the stone is in a difficult location because surgery is associated with high (10-30%) morbidity and mortality [6]. It involves mandatory lithotomy through gastrotomy, pylorotomy, and duodenotomy with/out extension of fistula followed by optional cholecystectomy and fistula repair or cholecystofistulectomy [3,6]. A long pyloroduodenotomy (as in our case), CGF, and suspicion of malignancy necessitates an occasional partial gastrectomy. Stone impacted in the distal duodenum is preferably manipulated towards the jejunum before extraction [6]. The distal bowel is checked for more stones [6]. Lithotomy is followed by staple or manual repair of fistula and cholecystectomy during the same or second operation after six weeks [1-3,5,6]. It is safer to not leave the wall of fistula or diseased gall bladder in the duodenum for fear of missing a cancer and leakage due to the infected granulation tissue in the fistula or ischemia of the retained gall bladder wall [23]. Graham’s patch and duodenojejunostomy help in safe duodenal closure [1,3,5,6]; while retrograde and subtotal cholecystectomy help in avoiding a bile duct injury [3,5]. When cholecystectomy is not possible, cholecystolithotomy during the index surgery may avoid recurrent gallstone ileus [11]. CCF is always staple closed with a sliver of the colonic wall (precaution for possible malignancy) or the colon is repaired after dividing the fistula.

Minimally invasive surgery (MIS) is favoured for CEF in specialist centres [1-6]. Meticulous dissection, skills in intracorporeal suturing, and staplers ensure safe and successful surgery [3,6]. The success of MIS for BS is gradually improving though numbers are small [22]. The rarity of the problem, an increasing number of successful endoscopies [22], conversion to laparotomy due to adhesions, bleeding, difficult intracorporal closure [6], direct laparotomy due to simultaneous Mirizzi Syndrome [24], or insufficient laparoscopy experience may be responsible for smaller numbers. There is no clear evidence at present on who is or is not an appropriate candidate for MIS in BS. Currently, it is probably the surgeon’s choice rather than parameters like stone size, location, multiple fistulae, Mirizzi syndrome, acute pancreatitis, or a combination of these factors.

Acute pancreatitis increases the difficulty in MIS due to added inflammation, makes duodenal closure difficult, and probably decreases the possibility of cholecystectomy [9,16]. In previously reported five cases of BS with acute pancreatitis, only one patient underwent laparoscopic enterolithotomy, fistula repair, and cholecystectomy and four patients had an open surgery; of the latter four, two patients had enterolithotomy and fistula repair with cholecystectomy while remaining two patients had only enterolithotomy but no cholecystectomy [9,14-17]. There isn’t much evidence on multiple fistulae and the role of MIS (again due to its rarity). While Becksac recommends open surgery [25], Costi supports MIS if the balance between advantages like early recovery and disadvantages like long hours or complications is maintained [4]. Despite these advances, there is still a role for nonoperative treatment of CEF when there is no emergency like BS, the emergency is resolved by endoscopy, or if the patient is unfit for surgery [6].

## Conclusions

Multiple CEF with BS and acute pancreatitis is a very rare presentation and is reported for the first time here. There is no established treatment approach for this complex situation. Evidence suggests that when the stone is <4 cm and located in the proximal duodenum, endoscopic retrieval followed by minimally invasive cholecystectomy and fistula repair is ideal. If endoscopy is unavailable, MIS can be the preferred mode of treatment. Skills in laparoscopic dissection, intracorporal suturing, and subtotal cholecystectomy are key to success with MIS. However, endoscopy and MIS is probably still not suitable for multiple fistulas, giant stone, and acute pancreatitis. In this situation, open surgery may be better. More evidence is needed to clarify the role of different treatment approaches. Till then the choice of treatment modality (endoscopy, MIS, open surgery) should be customised for better patient outcomes. 

## References

[1] Chowbey PK, Bandyopadhyay SK, Sharma A, Khullar R, Soni V, Baijal M (2006). Laparoscopic management of cholecystoenteric fistulas. J Laparoendosc Adv Surg Tech A.

[2] Li XY, Zhao X, Zheng P, Kao XM, Xiang XS, Ji W (2017). Laparoscopic management of cholecystoenteric fistula: a single-center experience. J Int Med Res.

[3] Huang SF, Han YH, Chen J, Zhang J, Huang H (2022). Surgical management of cholecystoenteric fistula in patients with and without gallstone ileus: an experience of 29 cases. Front Surg.

[4] Costi R, Randone B, Violi V (2009). Cholecystocolonic fistula: facts and myths. A review of the 231 published cases. J Hepatobiliary Pancreat Surg.

[5] Angrisani L, Corcione F, Tartaglia A (2001). Cholecystoenteric fistula (CF) is not a contraindication for laparoscopic surgery. Surg Endosc.

[6] Caldwell KM, Lee SJ, Leggett PL, Bajwa KS, Mehta SS, Shah SK (2018). Bouveret syndrome: current management strategies. Clin Exp Gastroenterol.

[7] Bouveret L (1896). Stenosis of the pylorus adherent to the calculous vesicle (Article in French). Rev Med.

[8] Tanwar S, Mawas A, Tutton M, O'Riordan D (2008). Successful endoscopic management of Bouveret's syndrome in a patient with cholecystoduodenocolic fistulae. Case Rep Gastroenterol.

[9] Poh WS, Wijesuriya R (2021). Case report - Bouveret's syndrome with pancreatitis: a rare combination. Int J Surg Case Rep.

[10] Ivanov I, Beuran M, Venter MD, Iftimie-Nastase I, Smarandache R, Popescu B, Bostina R (2012). Gallstone ileus after laparoscopic cholecystectomy. J Med Life.

[11] Stagnitti F, Stagnitti A, Tarcoveanu E (2021). Spontaneous biliary-enteric fistulas and associated complications: an overview. Chirurgia (Bucur).

[12] Glenn F, Reed C, Grafe WR (1981). Biliary enteric fistula. Surg Gynecol Obstet.

[13] Knol JA, Eckhauser FE (2002). Biliary fistulas. Shackelford’s Surgery of the Alimentary Tract, 5th Edition.

[14] Fenchel RF, Krige JE, Bornman PC (1999). Bouveret's syndrome complicated by acute pancreatitis. Dig Surg.

[15] Sica GS, Sileri P, Gaspari AL (2005). Laparoscopic treatment of Bouveret’s syndrome presenting as acute pancreatitis. JSLS.

[16] Zafar A, Ingham G, Jameel JK (2013). "Bouveret's syndrome" presenting with acute pancreatitis a very rare and challenging variant of gallstone ileus. Int J Surg Case Rep.

[17] Baloyiannis I, Symeonidis D, Koukoulis G, Zachari E, Potamianos S, Tzovaras G (2012). Complicated cholelithiasis: an unusual combination of acute pancreatitis and bouveret syndrome. Case Rep Gastroenterol.

[18] Sadovnikov I, Anthony M, Mushtaq R, Khreiss M, Gavini H, Arif-Tiwari H (2021). Role of magnetic resonance imaging in Bouveret's syndrome: a case report with review of the literature. Clin Imaging.

[19] Adnan AI, Vaz OP, Lapsia S, Sultana A, Ahmed MA (2022). Bouveret's syndrome: a case series and literature review on a gallstone disease causing gastric outlet obstruction. Cureus.

[20] Dumonceau JM, Devière J (2016). Novel treatment options for Bouveret's syndrome: a comprehensive review of 61 cases of successful endoscopic treatment. Expert Rev Gastroenterol Hepatol.

[21] Ong J, Swift C, Stokell BG (2020). Bouveret syndrome: a systematic review of endoscopic therapy and a novel predictive tool to aid in management. J Clin Gastroenterol.

[22] Swift C, Ong J, Zhou M, Stokell B, Al-Naeeb Y (2022). Post hoc validation of a tool that accurately predicts the outcome of endoscopic therapy in Bouveret syndrome. Gastroenterol Rep (Oxf).

[23] Prasad A, Foley RJ (1994). Laparoscopic management of cholecystocolic fistula. Br J Surg.

[24] Varshney VK, Hussain S, Selvakumar B, Vignesh N, Sureka B (2022). Mirizzi syndrome with Bouveret syndrome: a rare amalgam. Cureus.

[25] Beksac K, Erkan A, Kaynaroglu V (2016). Double incomplete internal biliary fistula: coexisting cholecystogastric and cholecystoduodenal fistula. Case Rep Surg.

